# 2,3-Diamino­pyridinium 4-hydroxy­benzoate

**DOI:** 10.1107/S1600536809020832

**Published:** 2009-06-06

**Authors:** Hoong-Kun Fun, Kasthuri Balasubramani

**Affiliations:** a X-ray Crystallography Unit, School of Physics, Universiti Sains Malaysia, 11800 Universiti Sains Malaysia, Penang, Malaysia

## Abstract

In the title compound, C_5_H_8_N_3_^+^·C_7_H_5_O_3_^−^, the pyridine N atom is protonated. In the 4-hydroxy­benzoate anion, the carboxyl­ate group is twisted slightly out of the benzene ring plane by an angle of 3.77 (5)°. The protonated N atom and one of the two amino groups are hydrogen-bonded to the 4-hydroxy­benzoate anion through a pair of N—H⋯O hydrogen bonds, forming an *R*_2_^2^(8) ring motif. The crystal structure is further stabilized by O—H⋯O and C—H⋯O hydrogen bonds and π–π inter­actions involving the pyridinium rings [centroid–centroid distance of 3.6277 (5) Å], leading to the formation of a three-dimensional network.

## Related literature

For general background to substituted pyridines, see: Pozharski *et al.* (1997 ▶); Katritzky *et al.* (1996 ▶). For details of hydrogen bonding, see: Jeffrey & Saenger (1991 ▶); Jeffrey (1997 ▶); Scheiner (1997 ▶). For hydrogen-bond motifs, see: Bernstein *et al.* (1995 ▶). For bond-length data, see: Allen *et al.* (1987 ▶). For the stability of the temperature controller used in the data collection, see: Cosier & Glazer (1986 ▶).
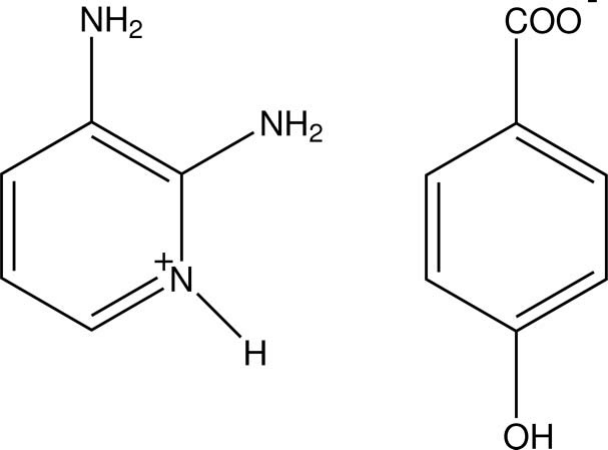


## Experimental

### 

#### Crystal data

C_5_H_8_N_3_^+^·C_7_H_5_O_3_^−^
*M* *_r_* = 247.25
Monoclinic, 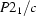

*a* = 10.2915 (2) Å
*b* = 11.4946 (2) Å
*c* = 11.0921 (2) Å
β = 112.644 (1)°
*V* = 1211.01 (4) Å^3^
*Z* = 4
Mo *K*α radiation
μ = 0.10 mm^−1^
*T* = 100 K
0.51 × 0.39 × 0.14 mm

#### Data collection

Bruker SMART APEXII CCD area-detector diffractometer
Absorption correction: multi-scan (*SADABS*; Bruker, 2005 ▶) *T*_min_ = 0.950, *T*_max_ = 0.986
25821 measured reflections
5296 independent reflections
4257 reflections with *I* > 2σ(*I*)
*R*_int_ = 0.034

#### Refinement

*R*[*F* ^2^ > 2σ(*F* ^2^)] = 0.049
*wR*(*F* ^2^) = 0.138
*S* = 1.04
5296 reflections
215 parameters
H atoms treated by a mixture of independent and constrained refinement
Δρ_max_ = 0.65 e Å^−3^
Δρ_min_ = −0.19 e Å^−3^

### 

Data collection: *APEX2* (Bruker, 2005 ▶); cell refinement: *SAINT* (Bruker, 2005 ▶); data reduction: *SAINT*; program(s) used to solve structure: *SHELXTL* (Sheldrick, 2008 ▶); program(s) used to refine structure: *SHELXTL*; molecular graphics: *SHELXTL*; software used to prepare material for publication: *SHELXTL* and *PLATON* (Spek, 2009 ▶).

## Supplementary Material

Crystal structure: contains datablocks global, I. DOI: 10.1107/S1600536809020832/ci2813sup1.cif

Structure factors: contains datablocks I. DOI: 10.1107/S1600536809020832/ci2813Isup2.hkl

Additional supplementary materials:  crystallographic information; 3D view; checkCIF report

## Figures and Tables

**Table 1:** Hydrogen-bond geometry (Å, °)

*D*—H⋯*A*	*D*—H	H⋯*A*	*D*⋯*A*	*D*—H⋯*A*
N1—H1*N*1⋯O2	0.89 (2)	1.903 (15)	2.7874 (9)	173 (1)
N2—H2*N*2⋯O3	0.89 (1)	1.898 (14)	2.7843 (9)	176 (1)
N2—H1*N*2⋯O2^i^	0.87 (1)	2.014 (15)	2.8689 (9)	168 (1)
N3—H1*N*3⋯O2^i^	0.91 (2)	2.071 (16)	2.9790 (10)	174 (1)
N3—H2*N*3⋯O3^ii^	0.89 (2)	2.057 (15)	2.9285 (10)	166 (1)
O1—H1*O*1⋯O3^iii^	0.90 (2)	1.775 (19)	2.6595 (8)	168 (2)
C2—H2⋯O3^iii^	1.00 (1)	2.500 (14)	3.2104 (10)	128 (1)

Symmetry codes: (i) 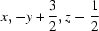; (ii) 
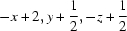
; (iii) 
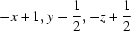
.

**Fig. 1.**
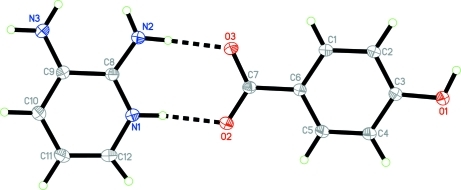
The molecular structure of the title compound, showing 50% probability displacement ellipsoids and the atom numbering scheme. Dashed lines indicate hydrogen bonds.

**Fig. 2.**
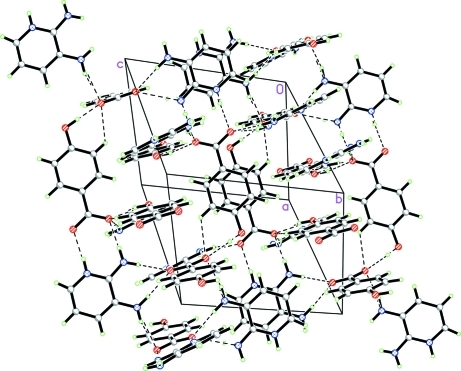
Crystal packing of the title compound, showing a part of the three-dimensional network. Hydrogen bonds are shown as dashed lines.

## supplementary crystallographic information

### Comment

Pyridine and its derivatives play an important role in heterocyclic chemistry (Pozharski *et al.*, 1997; Katritzky *et al.*, 1996)). Pyridine and its substituted derivatives are often involved in hydrogen-bond interactions (Jeffrey & Saenger, 1991; Jeffrey, 1997;Scheiner, 1997). Since our aim is to study some interesting hydrogen-bonding interactions, the crystal structure of the title compound is presented here.

The asymmetric unit of the title compound (Fig 1), contains one 2,3-diaminopyridinium cation and one 4-hydroxybenzoate anion. The bond lengths (Allen *et al.*, 1987) and angles are normal. The 2,3-diaminopyridinium cation is planar to within ±0.015 (1) Å. The protonation of N1 atom resulted in a slight increase in the C8—N1—C12 angle [123.47 (7)°]. In the anion, the carboxylate group is twisted slightly away from the attached ring; the dihedral angle between C1-C6 and O2/O3/C7/C6 planes is 3.77 (5)°.

In the crystal packing (Fig. 2), the protonated N1 atom and the 2-amino group (N2) is hydrogen-bonded to the carboxylate oxygen atoms (O2 and O3) *via* a pair of N—H···O hydrogen bonds forming a ring motif *R*_2_^2^(8) (Bernstein *et al.*, 1995). The amino groups (N2 and N3) are involved in N—H···O hydrogen bonding interactions to form a *R*^1^_2_(7) ring motif. The hydroxyl group hydrogen atom is also hydrogen-bonded to the carboxylate oxygen atom through O—H···O hydrogen bonds. Moreover O—H···O and C—H···O hydrogen bonds form a *R*^1^_2_(6) ring motif (Table 1 and Fig 2). The crystal structure is further stabilized by π-π stacking interactions between the pyridinium rings (C8—C12/N1) at (x, y, z) and (2-x, 2-y, 1-z), with a centroid-to-centroid distance of 3.6277 (5) Å.

### Experimental

Hot methanol solutions (20 ml) of 2,3-diaminopyridine (27 mg, Aldrich) and 4-hydroxybenzoic acid (35 mg, Merck) were mixed and warmed over a heating magnetic stirrer for 5 minutes. The resulting solution was allowed to cool slowly at room temperature. Crystals of the title compound appeared from the mother liquor after a few days.

### Refinement

All the H atoms were located in a difference Fourier map and allowed to refine freely [N–H = 0.86–0.91 Å, C–H = 0.95–1.01 (15)Å and O–H = 0.89 (18) Å]

### Crystal data

**Table:**

C_5_H_8_N_3_^+^·C_7_H_5_O_3_^−^	*F*(000) = 520
*M**_r_* = 247.25	*D*_x_ = 1.356 Mg m^−^^3^
Monoclinic, *P*2_1_/*c*	Mo *K*α radiation, λ = 0.71073 Å
Hall symbol: -P 2ybc	Cell parameters from 9979 reflections
*a* = 10.2915 (2) Å	θ = 2.7–37.8°
*b* = 11.4946 (2) Å	µ = 0.10 mm^−^^1^
*c* = 11.0921 (2) Å	*T* = 100 K
β = 112.644 (1)°	Plate, brown
*V* = 1211.01 (4) Å^3^	0.51 × 0.39 × 0.14 mm
*Z* = 4	

### Data collection

**Table:**

Bruker SMART APEXII CCD area-detector diffractometer	5296 independent reflections
Radiation source: fine-focus sealed tube	4257 reflections with *I* > 2σ(*I*)
graphite	*R*_int_ = 0.034
φ and ω scans	θ_max_ = 35.0°, θ_min_ = 2.1°
Absorption correction: multi-scan (*SADABS*; Bruker, 2005)	*h* = −16→16
*T*_min_ = 0.950, *T*_max_ = 0.986	*k* = −16→18
25821 measured reflections	*l* = −17→17

### Refinement

**Table:**

Refinement on *F*^2^	Primary atom site location: structure-invariant direct methods
Least-squares matrix: full	Secondary atom site location: difference Fourier map
*R*[*F*^2^ > 2σ(*F*^2^)] = 0.049	Hydrogen site location: inferred from neighbouring sites
*wR*(*F*^2^) = 0.138	H atoms treated by a mixture of independent and constrained refinement
*S* = 1.04	*w* = 1/[σ^2^(*F*_o_^2^) + (0.0817*P*)^2^ + 0.1673*P*] where *P* = (*F*_o_^2^ + 2*F*_c_^2^)/3
5296 reflections	(Δ/σ)_max_ = 0.001
215 parameters	Δρ_max_ = 0.65 e Å^−^^3^
0 restraints	Δρ_min_ = −0.18 e Å^−^^3^

### Special details

**Table:**

Experimental. The crystal was placed in the cold stream of an Oxford Cyrosystems Cobra open-flow nitrogen cryostat (Cosier & Glazer, 1986) operating at 100.0 (1) K.
Geometry. All s.u.'s (except the s.u. in the dihedral angle between two l.s. planes) are estimated using the full covariance matrix. The cell s.u.'s are taken into account individually in the estimation of s.u.'s in distances, angles and torsion angles; correlations between s.u.'s in cell parameters are only used when they are defined by crystal symmetry. An approximate (isotropic) treatment of cell s.u.'s is used for estimating s.u.'s involving l.s. planes.
Refinement. Refinement of F^2^ against ALL reflections. The weighted R-factor wR and goodness of fit S are based on F^2^, conventional R-factors R are based on F, with F set to zero for negative F^2^. The threshold expression of F^2^ > σ(F^2^) is used only for calculating R-factors(gt) etc. and is not relevant to the choice of reflections for refinement. R-factors based on F^2^ are statistically about twice as large as those based on F, and R- factors based on ALL data will be even larger.

### Fractional atomic coordinates and isotropic or equivalent isotropic displacement parameters (Å^2^)

**Table:**

	*x*	*y*	*z*	*U*_iso_*/*U*_eq_	
O1	0.45467 (8)	0.16900 (6)	0.43336 (6)	0.02563 (15)	
O2	0.84786 (7)	0.61461 (5)	0.52034 (5)	0.02074 (13)	
O3	0.73136 (6)	0.62156 (5)	0.30541 (5)	0.01784 (12)	
N1	1.01845 (8)	0.79427 (6)	0.49674 (6)	0.01857 (14)	
N2	0.87767 (8)	0.81703 (7)	0.27844 (7)	0.02195 (15)	
N3	1.05767 (9)	0.99458 (8)	0.25977 (7)	0.02666 (17)	
C1	0.57827 (9)	0.42070 (7)	0.30431 (7)	0.01757 (14)	
C2	0.50323 (9)	0.32084 (7)	0.30618 (7)	0.01879 (15)	
C3	0.52248 (9)	0.26810 (7)	0.42538 (7)	0.01899 (15)	
C4	0.61617 (11)	0.31656 (8)	0.54174 (8)	0.02518 (18)	
C5	0.69083 (10)	0.41624 (8)	0.53881 (7)	0.02324 (17)	
C6	0.67313 (8)	0.46970 (7)	0.41995 (7)	0.01604 (14)	
C7	0.75521 (8)	0.57533 (6)	0.41592 (7)	0.01540 (14)	
C8	0.99181 (9)	0.84838 (7)	0.38181 (7)	0.01678 (14)	
C9	1.08674 (9)	0.93716 (7)	0.37590 (7)	0.01803 (15)	
C10	1.20049 (9)	0.96332 (8)	0.48910 (8)	0.02092 (16)	
C11	1.22331 (9)	0.90438 (8)	0.60700 (8)	0.02314 (17)	
C12	1.13117 (10)	0.82008 (8)	0.60867 (8)	0.02224 (17)	
H1	0.5647 (15)	0.4565 (11)	0.2226 (13)	0.031 (3)*	
H2	0.4376 (14)	0.2854 (12)	0.2229 (13)	0.029 (3)*	
H4	0.6280 (18)	0.2759 (15)	0.6232 (17)	0.051 (4)*	
H5	0.7615 (17)	0.4479 (13)	0.6225 (15)	0.039 (4)*	
H10	1.2669 (14)	1.0227 (12)	0.4848 (12)	0.028 (3)*	
H11	1.3091 (16)	0.9246 (13)	0.6883 (14)	0.039 (4)*	
H12	1.1341 (17)	0.7745 (14)	0.6809 (15)	0.043 (4)*	
H1N1	0.9588 (15)	0.7391 (12)	0.4987 (13)	0.031 (3)*	
H1N2	0.8601 (17)	0.8454 (12)	0.2011 (15)	0.033 (3)*	
H2N2	0.8275 (15)	0.7565 (12)	0.2854 (13)	0.027 (3)*	
H1N3	0.9924 (17)	0.9660 (13)	0.1840 (15)	0.040 (4)*	
H2N3	1.1284 (16)	1.0365 (12)	0.2548 (14)	0.034 (3)*	
H1O1	0.3995 (19)	0.1445 (15)	0.3531 (18)	0.051 (5)*	

### Atomic displacement parameters (Å^2^)

**Table:**

	*U*^11^	*U*^22^	*U*^33^	*U*^12^	*U*^13^	*U*^23^
O1	0.0299 (3)	0.0248 (3)	0.0172 (3)	−0.0123 (2)	0.0034 (2)	0.0013 (2)
O2	0.0238 (3)	0.0217 (3)	0.0141 (2)	−0.0059 (2)	0.0044 (2)	−0.00153 (19)
O3	0.0188 (3)	0.0178 (3)	0.0149 (2)	0.0002 (2)	0.00434 (19)	0.00308 (19)
N1	0.0200 (3)	0.0194 (3)	0.0148 (3)	−0.0020 (2)	0.0051 (2)	0.0018 (2)
N2	0.0232 (3)	0.0234 (3)	0.0154 (3)	−0.0072 (3)	0.0031 (2)	0.0021 (2)
N3	0.0284 (4)	0.0324 (4)	0.0171 (3)	−0.0118 (3)	0.0065 (3)	0.0037 (3)
C1	0.0190 (3)	0.0187 (3)	0.0131 (3)	−0.0014 (3)	0.0041 (2)	0.0005 (2)
C2	0.0198 (4)	0.0205 (3)	0.0131 (3)	−0.0037 (3)	0.0031 (2)	−0.0007 (2)
C3	0.0202 (4)	0.0199 (3)	0.0150 (3)	−0.0046 (3)	0.0047 (3)	−0.0001 (2)
C4	0.0316 (5)	0.0273 (4)	0.0130 (3)	−0.0114 (3)	0.0046 (3)	0.0008 (3)
C5	0.0286 (4)	0.0251 (4)	0.0131 (3)	−0.0099 (3)	0.0049 (3)	−0.0015 (3)
C6	0.0173 (3)	0.0167 (3)	0.0131 (3)	−0.0016 (2)	0.0048 (2)	−0.0007 (2)
C7	0.0167 (3)	0.0152 (3)	0.0140 (3)	0.0013 (2)	0.0055 (2)	−0.0002 (2)
C8	0.0177 (3)	0.0174 (3)	0.0143 (3)	−0.0003 (3)	0.0052 (2)	0.0001 (2)
C9	0.0183 (3)	0.0204 (3)	0.0155 (3)	−0.0023 (3)	0.0066 (2)	0.0000 (2)
C10	0.0192 (4)	0.0249 (4)	0.0178 (3)	−0.0044 (3)	0.0062 (3)	−0.0010 (3)
C11	0.0200 (4)	0.0294 (4)	0.0166 (3)	−0.0029 (3)	0.0032 (3)	−0.0007 (3)
C12	0.0230 (4)	0.0262 (4)	0.0145 (3)	−0.0014 (3)	0.0038 (3)	0.0023 (3)

### Geometric parameters (Å, °)

**Table:**

O1—C3	1.3565 (10)	C2—C3	1.3976 (11)
O1—H1O1	0.897 (18)	C2—H2	0.997 (13)
O2—C7	1.2666 (9)	C3—C4	1.3956 (11)
O3—C7	1.2702 (9)	C4—C5	1.3869 (12)
N1—C8	1.3484 (10)	C4—H4	0.982 (17)
N1—C12	1.3661 (11)	C5—C6	1.4014 (11)
N1—H1N1	0.888 (15)	C5—H5	1.001 (15)
N2—C8	1.3369 (10)	C6—C7	1.4896 (11)
N2—H1N2	0.869 (15)	C8—C9	1.4316 (11)
N2—H2N2	0.887 (14)	C9—C10	1.3805 (11)
N3—C9	1.3739 (10)	C10—C11	1.4100 (12)
N3—H1N3	0.911 (16)	C10—H10	0.981 (14)
N3—H2N3	0.892 (15)	C11—C12	1.3608 (13)
C1—C2	1.3881 (11)	C11—H11	1.017 (15)
C1—C6	1.3969 (10)	C12—H12	0.948 (15)
C1—H1	0.955 (13)		
			
C3—O1—H1O1	110.1 (11)	C4—C5—H5	119.3 (8)
C8—N1—C12	123.47 (7)	C6—C5—H5	119.9 (9)
C8—N1—H1N1	117.5 (9)	C1—C6—C5	118.61 (7)
C12—N1—H1N1	119.1 (9)	C1—C6—C7	120.33 (6)
C8—N2—H1N2	121.7 (10)	C5—C6—C7	121.05 (7)
C8—N2—H2N2	119.1 (9)	O2—C7—O3	122.08 (7)
H1N2—N2—H2N2	118.2 (13)	O2—C7—C6	119.94 (6)
C9—N3—H1N3	120.6 (10)	O3—C7—C6	117.96 (6)
C9—N3—H2N3	115.4 (9)	N2—C8—N1	118.57 (7)
H1N3—N3—H2N3	117.8 (13)	N2—C8—C9	122.86 (7)
C2—C1—C6	121.05 (7)	N1—C8—C9	118.57 (7)
C2—C1—H1	119.4 (8)	N3—C9—C10	123.40 (8)
C6—C1—H1	119.5 (8)	N3—C9—C8	118.64 (7)
C1—C2—C3	119.72 (7)	C10—C9—C8	117.92 (7)
C1—C2—H2	120.4 (8)	C9—C10—C11	121.27 (8)
C3—C2—H2	119.9 (8)	C9—C10—H10	118.1 (8)
O1—C3—C4	117.67 (7)	C11—C10—H10	120.6 (8)
O1—C3—C2	122.47 (7)	C12—C11—C10	119.12 (8)
C4—C3—C2	119.85 (7)	C12—C11—H11	121.5 (8)
C5—C4—C3	119.99 (7)	C10—C11—H11	119.4 (8)
C5—C4—H4	122.8 (10)	C11—C12—N1	119.65 (7)
C3—C4—H4	117.2 (10)	C11—C12—H12	127.5 (10)
C4—C5—C6	120.78 (7)	N1—C12—H12	112.8 (10)
			
C6—C1—C2—C3	−0.01 (13)	C5—C6—C7—O3	−178.02 (8)
C1—C2—C3—O1	−178.47 (8)	C12—N1—C8—N2	−179.40 (8)
C1—C2—C3—C4	0.46 (14)	C12—N1—C8—C9	0.41 (12)
O1—C3—C4—C5	178.40 (9)	N2—C8—C9—N3	1.55 (13)
C2—C3—C4—C5	−0.57 (15)	N1—C8—C9—N3	−178.25 (8)
C3—C4—C5—C6	0.24 (15)	N2—C8—C9—C10	179.31 (8)
C2—C1—C6—C5	−0.32 (13)	N1—C8—C9—C10	−0.49 (12)
C2—C1—C6—C7	178.59 (7)	N3—C9—C10—C11	177.91 (9)
C4—C5—C6—C1	0.21 (14)	C8—C9—C10—C11	0.26 (13)
C4—C5—C6—C7	−178.70 (9)	C9—C10—C11—C12	0.06 (14)
C1—C6—C7—O2	−175.21 (8)	C10—C11—C12—N1	−0.16 (14)
C5—C6—C7—O2	3.67 (12)	C8—N1—C12—C11	−0.08 (13)
C1—C6—C7—O3	3.09 (11)		

### Hydrogen-bond geometry (Å, °)

**Table:**

*D*—H···*A*	*D*—H	H···*A*	*D*···*A*	*D*—H···*A*
N1—H1N1···O2	0.89 (2)	1.903 (15)	2.7874 (9)	173 (1)
N2—H2N2···O3	0.89 (1)	1.898 (14)	2.7843 (9)	176 (1)
N2—H1N2···O2^i^	0.87 (1)	2.014 (15)	2.8689 (9)	168 (1)
N3—H1N3···O2^i^	0.91 (2)	2.071 (16)	2.9790 (10)	174 (1)
N3—H2N3···O3^ii^	0.89 (2)	2.057 (15)	2.9285 (10)	166 (1)
O1—H1O1···O3^iii^	0.90 (2)	1.775 (19)	2.6595 (8)	168 (2)
C2—H2···O3^iii^	1.00 (1)	2.500 (14)	3.2104 (10)	128 (1)

Symmetry codes: (i) *x*, −*y*+3/2, *z*−1/2; (ii) −*x*+2, *y*+1/2, −*z*+1/2; (iii) −*x*+1, *y*−1/2, −*z*+1/2.
